# Multiplexed reverse transcription real-time polymerase chain reaction for simultaneous detection of Mayaro, Oropouche, and Oropouche-like viruses

**DOI:** 10.1590/0074-02760160062

**Published:** 2017-07

**Authors:** Felipe Gomes Naveca, Valdinete Alves do Nascimento, Victor Costa de Souza, Bruno Tardelli Diniz Nunes, Daniela Sueli Guerreiro Rodrigues, Pedro Fernando da Costa Vasconcelos

**Affiliations:** 1 Fundação Oswaldo Cruz-Fiocruz, Instituto Leônidas e Maria Deane, Manaus, AM, Brasil; 2 Ministério da Saúde, Secretaria de Vigilância em Saúde, Instituto Evandro Chagas, Ananindeua, PA, Brasil; 3 Unversidade do Estado do Pará, Belém, PA, Brasil

**Keywords:** real-time PCR, Mayaro virus, Oropouche virus, Amazon

## Abstract

We describe a sensitive method for simultaneous detection of Oropouche and Oropouche-like viruses carrying the Oropouche S segment, as well as the Mayaro virus, using a multiplexed one-step reverse transcription real-time polymerase chain reaction (RT-qPCR). A chimeric plasmid containing both Mayaro and Oropouche targets was designed and evaluated for the *in vitro* production of transcribed RNA, which could be easily used as a non-infectious external control. To track false-negative results due to PCR inhibition or equipment malfunction, the MS2 bacteriophage was also included in the multiplex assay as an internal positive control. The specificity of the multiplex assay was evaluated by Primer-Blast analysis against the entire GenBank database, and further against a panel of 17 RNA arboviruses. The results indicated an accurate and highly sensitive assay with amplification efficiency greater than 98% for both targets, and a limit of detection between two and 20 copies per reaction. We believe that the assay described here will provide a tool for Mayaro and Oropouche virus detection, especially in areas where differential diagnosis of Dengue, Zika and Chikungunya viruses should be performed.

Arthropod-borne viruses (arboviruses) are among the most important causes of emerging or re-emerging infectious diseases worldwide. Besides the great concern regarding Dengue, Zika and Chikungunya infections, epidemiological data showing the emergence of two other arboviruses known as Mayaro (MAYV) and Oropouche (OROV), suggest that these viruses are worthy of special attention, specifically in the northern region of South America (Mourão et al. 2009, Vasconcelos et al. 2009, Bastos et al. 2012, Cardoso et al. 2015). In the present study, we describe a sensitive and specific method for one-step reverse transcription real-time polymerase chain reaction (RT-qPCR) for the detection of MAYV, OROV, and other OROV-like viruses carrying the OROV S segment, coupled with an internal control.

All available and complete GenBank genome sequences for MAYV or that of the full S segment for OROV were used for two different nucleotide alignments, one for each target virus, using ClustalX software (Jeanmougin et al. 1998). Conserved regions were chosen and analysed for primer and probe design using Primer Express 3.0 (Applied Biosystems, Thermo Scientific, CA, USA) with the default parameters. Two primer and probe sets were designed, according to the lower penalties score in Primer Express, targeting the NSP1 coding sequence of MAYV and the S segment of OROV. Selected primer sets were also submitted to primer-blast analysis (Ye et al. 2012) of the entire GenBank database under default parameters, except the minimum PCR product size (50 bp). Primer pair specificity for the database parameter was set to ‘nr’ and the organism parameter was set to ‘all’. A third TaqMan MGB probe was designed as described above, targeting the coat protein (cp) gene of the *Enterobacteria* phage MS2 (ATCC 15597B1), used as the positive internal control (Table).

**TABLE:** : Oligonucleotides designed in this study

Oligo	Sequence	Start	Stop
MAYV_FNF	5’ CACGGACMTTTTGCCTTCA 3’	465	483
MAYV_FNR	5’ AGACTGCCACCTCTGCTKGAG 3’	524	504
MAYV_FNP	5’(VIC) ACAGATCAGACATGCAGG 3’	485	502
OROV_FNF	5’ TCCGGAGGCAGCATATGTG 3’	98	116
OROV_FNR	5’ ACAACACCAGCATTGAGCACTT 3’	160	139
OROV_FNP	5’(FAM) CATTTGAAGCTAGATACGG 3’	118	136
MS2_IC_FNF	5’ GCGCAGAATCGCAAATACA 3’	1494	1512
MS2_IC_FNR	5’ CAACAGTCTGGGTTGCCACTT 3’	1554	1534
MS2_IC_FNP	5’(NED) ATCAAAGTCGAGGTGCC 3’	1515	1531

Start/Stop numbers refers to the nucleotide position of Mayaro (MAYV) and Oropouche (OROV) S segment, and the *Enterobacteria* phage MS2 GenBank reference sequences (NC_003417.1, NC_005777.1, and NC_001417.2, respectively). All probes are TaqMan Minor Groove Binding (MGB) type with the fluorophores FAM, VIC, or NED as reporters and a non-fluorescent quencher. IDT DNA Technology synthesised all desalted primers used in this study with no further purification, whereas Applied Biosystems supplied the probes. The last digit in each oligo name was used to identify the forward (F) and reverse (R) primers, as well as the probes (P).

After choosing the primer and probe sets, a synthetic positive external control encompassing both MAYV and OROV target regions (Fig. 1) was ordered as a custom-made plasmid named pOROV_MAYV form IDT DNA Technology (IA, USA). The RT-qPCR target was located downstream of a T7 RNA polymerase promoter site, which allowed us to produce chimeric RNA molecules containing both targets with TranscriptAid^TM^ T7 High Yield Transcription Kit according to manufactures’ instructions (Fermentas UAB, Thermo Scientific, Vilnius, Lithuania). Before the *in vitro* transcription of RNA, the insert and the T7 promoter site were PCR amplified (amplicon size, 925 bp) to limit the template size, for enhancement of *in vitro* RNA transcription.

**Fig. 1:**
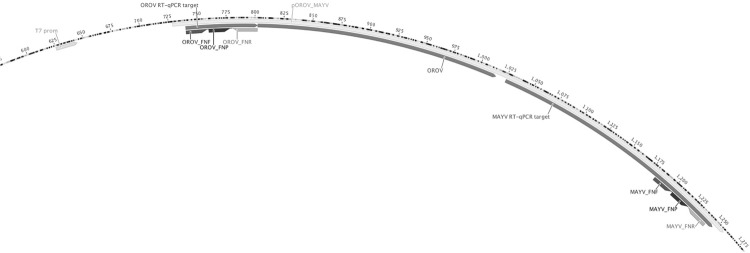
: chimeric plasmid pOROV_MAYV. The pOROV_MAYV plasmid contains both Mayaro (MAYV) and Oropouche (OROV) targets in the context for *in vitro* RNA production from the T7 promoter site. The forward and reverse primer binding sites, as well as the probe binding sites are represented by a dark grey, light grey, and a black arrow, respectively. The pOROV_MAYV plasmid was ordered form IDT DNA Technology.

The generated amplicons were precipitated with molecular biology grade polyethylene glycol 8000 (20% w/v; Promega, WI, USA) for primers and dNTP removal. DNase digested *in vitro* transcribed RNA was quantified with a Qubit® RNA HS Assay Kit using a Qubit® 2.0 Fluorometer (Invitrogen, Thermo Scientific, CA, USA). RNA quantitation and its sequence were used to calculate the exact target copy numbers with the aid of the endmemo server (endmemo.com/bio/dnacopynum.php). The *in vitro* transcribed RNA was used in all optimisation steps of this protocol, including primer and probe titration. We used the TaqMan® Fast Virus 1-Step master mix (Applied Biosystems) for RT-qPCR amplification with the recommended cycling parameters, 50ºC for 5 min for reverse transcription, 95ºC for 20 s for RT inactivation/initial denaturation, followed by 45 cycles of 95ºC for 3 s and 60ºC for 30 s. RNA (2 μL) was used as a template in a 20 µL reaction, and all assays were performed using the StepOnePlus Real-Time PCR System (Applied Biosystems). The assay amplification efficiency was calculated by the standard curve method (Svec et al. 2015) using a 10-fold, 8-log, serial dilution starting at 2 × 10^8^ RNA copies/µL, in duplicate.

Primer and probe specificity were evaluated against a panel of RNA arboviruses from the Brazilian National Reference Laboratory for Arbovirus at the Instituto Evandro Chagas. Viral RNA from MAYV and OROV, as well as 15 other arboviruses, from three different families were extracted from previously infected suckling mouse brain or liver with Trizol LS (Invitrogen), as recommended by the manufacturer. These included Flaviviridae (Dengue virus serotypes 1, 2, 3, and 4 and Ilheus, Rocio, Saint Louis Encephalitis, and Zika viruses), Togaviridae (Chikungunya, Trocara, and Western Equine Encephalitis viruses), and Bunyaviridae (Caraparu, Catu, Icoaraci, Jatobal, Tacaiuma, Trocara, and Utinga viruses).

After optimisation to determine the best primer and probe concentrations, we used 300 nM of each primer and 100 nM of each TaqMan MGB Probe. According to the standard curve results, and considering a Ct value lower than 38 as positive, the multiplex assay had a limit of detection between 2-20 copies (Mean Ct values, 34.9-38.3 and 34.8-38.1 for MAYV and OROV, respectively). No considerable loss in sensitivity was observed in the multiplex format in comparison to single reactions. In fact, both MAYV and OROV amplification efficiencies showed excellent results and reproducibility in the multiplex format (Fig. 2).

**Fig. 2:**
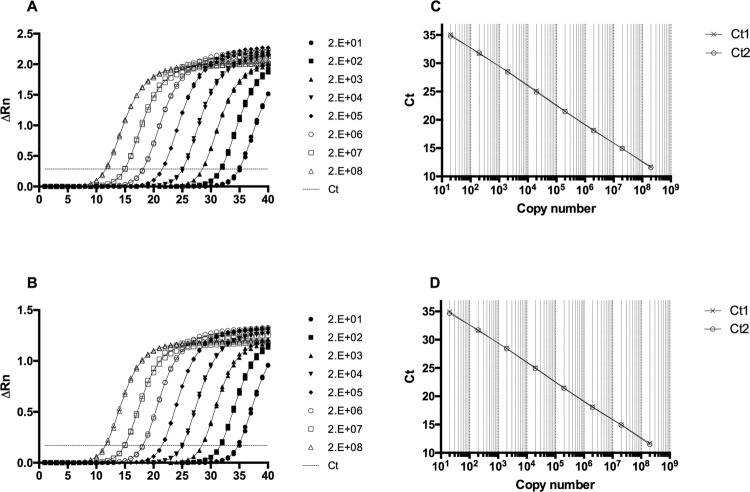
: reverse transcription real-time polymerase chain reaction (RT-qPCR) with serial dilutions of the chimeric *in vitro* transcribed RNA containing both Mayaro (MAYV) and Oropouche (OROV) targets. Amplification plots for MAYV (A) and OROV (B), and linear regression for MAYV (C) and OROV (D) for ten-fold, 8-log, dilutions from 20 to 2E+08 copies, in duplicate. PCR efficiency in the multiplex assay was calculated with StepOnePlus Software v2.2 to be 98.642% [slope: -3.355, R2: 1] for MAYV, and 99.181% [slope: -3.341, R2: 1] for OROV. Ct = cycle threshold. Fluorescence values were exported to MS Excel and plotted using GraphPad Prism 6.0.

As expected, no amplification was observed when the supernatant of uninfected cell lines, commonly used for arboviral isolation (C6/36 and Vero), or other arboviral RNA (those previously specified) were used as templates for the RT-qPCR reaction, with the exception of the Jatobal virus, an OROV reassortant virus, that was amplified with the OROV set. In those negative samples, only the MS2 amplification curve was consistently observed.

Bunyaviruses such as OROV possess a segmented genome encompassing three RNA segments referred to as L (large), M (medium), and S (small). This characteristic, also found in influenza and reoviruses, results in evolution through segment reassortment, which might culminate in the generation of a new virus (Briese et al. 2013). This phenomenon was observed in at least four bunyaviruses that have the S segment of OROV. The reassortant OROV-like virus Jatobal that has an S segment with more than 95% similarity to that of the OROV prototype (Saeed et al. 2001). In addition Madre de Dios virus (Ladner et al. 2014), Iquitos virus (Aguilar et al. 2011), and Perdoes virus (Tilston-Lunel et al. 2015) share S and L segments with OROV. These OROV-like viruses could also be amplified by the primer and probe sets designed in this study. Primer-Blast analysis with the OROV primer set returned 104 OROV sequences having a perfect match, and another 10 sequences of Jatobal, Iquitos, Madre de Dios, and Perdoes virus without a mismatch. Another 36 OROV sequences showed one or two mismatches related to the primer sequences. Regarding the MAYV primer set, primer-Blast analysis returned 37 sequences with a perfect match and 12 with one mismatch (Supplementary data). For the OROV set, as well as for the MAYV set, non-specific matches were not observed.

MAYV is a member of the *Alphavirus* genus in the Togaviridae family; it is closely related to the Chikungunya virus, and since its initial description, MAYV has been associated with cases of self-limited illness, presenting fever, rash, and severe arthralgia as the most common symptoms (Azevedo et al. 2009, Abad-Franch et al. 2012, Mourão et al. 2012). OROV belongs to the *Orthobunyavirus* genus, in the Bunyaviridae family, and is often associated with outbreaks of febrile acute Dengue-like disease. For several years, Oropouche fever was considered the second most common arboviral disease in Brazil, surpassed only by dengue fever (Figueiredo 2007, Vasconcelos et al. 2011). Nevertheless, the current Brazilian epidemiological scenario indicates that Zika virus infections have assumed this position. Therefore, in the context of different arboviruses cocirculating, the appropriate diagnosis of acute febrile illness, based only on clinical examination, becomes even more difficult.

Nucleic acid amplification tests (NAATs) have revamped diagnostics of infectious diseases, not only through its inherent superior sensitivity and specificity (when properly optimised), but also because of its feasibility in epidemic situations. Moreover, critical information required for their development (i.e., pathogen nucleotide sequences) is usually unrestrictedly available in public databases. Thus, the necessity for fast, sensitive, and reliable molecular tests for emerging arboviruses led us to develop the protocol described in this study.

In this study, we evaluated the use of a multiplexed RT-qPCR protocol with positive and negative viral controls. Viral RNA was obtained from cell supernatants, mouse brain, or liver, in addition to *in vitro* transcribed RNA, but no human clinical samples were analysed. Detection of viral RNA direct from clinical specimens is usually more challenging due to lower viral loads in comparison to what is found in the supernants of infected cells and in the tissues of inoculated mice, however, the sensitivity observed for this optimised protocol (< 20 copies per reaction) seems to be sufficient for this application. In fact, this protocol is already being used in another study to detect human Oropouche cases in the state of Amazonas (R Figueiredo, personal communication).

In conclusion, we have developed an accurate, reproducible, and highly sensitive one-step RT-qPCR protocol for the detection of two emerging arboviruses. We believe this protocol, together with other NAATs already published, will help reduce the number of misdiagnoses of acute febrile cases that are under the dengue umbrella.
