# Supplementary material for: Multiplexed reverse transcription real-time polymerase chain reaction for simultaneous detection of Mayaro, Oropouche, and Oropouche-like viruses
Source: Mem Inst Oswaldo Cruz. 2017 Jul;112(7):510–3. doi: 10.1590/0074-02760160062 (PMC5452489; doi:10.1590/0074-02760160062)
Supplement: Supplementary file 1 [file 0074-0276-mioc-112-7-0510-suppl01.pdf]

 Mem Inst Oswaldo Cruz, Rio de Janeiro, Vol. 112(7): 1-4, July 2017

Primer-Blast analysis of Oropouche (OROV) and Mayaro (MAYV) primers

OROV 100% match

KP691623.1 Oropouche virus isolate BeH759620
KP691632.1 Oropouche virus isolate BeH759146 segment S, complete sequence
KP052852.1 Oropouche virus strain BeAn19991 segment S, complete sequence
KC759130.1 Oropouche virus strain GML-444479 segment S nucleocapsid protein (N) and nonstructural protein (NS) genes, complete cds
KC759127.1 Oropouche virus strain IQT-1690 segment S nucleocapsid protein (N) and nonstructural protein (NS) genes, complete cds
HM470142.1 Oropouche virus strain BeH505805 nucleocapsid protein gene, partial cds
HM470141.1 Oropouche virus strain BeH505768 nucleocapsid protein gene, partial cds
HM470140.1 Oropouche virus strain BeH505764 nucleocapsid protein gene, partial cds
HM470139.1 Oropouche virus strain BeH498913 nucleocapsid protein gene, partial cds
HM470138.1 Oropouche virus strain BeH708717 nucleocapsid protein gene, complete cds
HM470136.1 Oropouche virus strain BeH707157 nucleocapsid protein gene, complete cds
HM470135.1 Oropouche virus strain BeH708139 nucleocapsid protein gene, partial cds
HM470134.1 Oropouche virus strain BeH706893 nucleocapsid protein gene, complete cds
HM470133.1 Oropouche virus strain BeH706890 nucleocapsid protein gene, complete cds
HM470123.1 Oropouche virus strain BeH532422 nucleocapsid protein gene, partial cds
HM470122.1 Oropouche virus strain BeH532314 nucleocapsid protein gene, partial cds
HM470117.1 Oropouche virus strain BeAr366927 nucleocapsid protein gene, partial cds
HM470115.1 Oropouche virus strain BeH355186 nucleocapsid protein gene, complete cds
HM470113.1 Oropouche virus strain BeAr271708 nucleocapsid protein gene, partial cds
HM470111.1 Oropouche virus strain BeAr136921 nucleocapsid protein gene, complete cds
HM470105.1 Oropouche virus strain BeH472433 nucleocapsid protein gene, partial cds
HM470102.1 Oropouche virus strain BeH543100 nucleocapsid protein gene, partial cds
HM470137.1 Oropouche virus strain BeH707287 nucleocapsid protein gene, partial cds
HM470114.1 Oropouche virus strain BeH355173 nucleocapsid protein gene, partial cds
HQ830491.1 Oropouche virus isolate H759044 nucleoprotein (N) and non-structural protein (NSs) genes, complete cds
HQ830489.1 Oropouche virus isolate H759042 nucleoprotein (N) and non-structural protein (NSs) genes, complete cds
HQ830488.1 Oropouche virus isolate H759041 nucleoprotein (N) and non-structural protein (NSs) genes, complete cds
HQ830486.1 Oropouche virus isolate H759018 nucleoprotein (N) and non-structural protein (NSs) genes, complete cds
HQ830485.1 Oropouche virus isolate H759562 nucleoprotein (N) and non-structural protein (NSs) genes, complete cds
HQ830484.1 Oropouche virus isolate H759038 nucleoprotein (N) and non-structural protein (NSs) genes, complete cds
HQ830483.1 Oropouche virus isolate H75955 nucleoprotein (N) and non-structural protein (NSs) genes, complete cds
HQ830481.1 Oropouche virus isolate H759541 nucleoprotein (N) and non-structural protein (NSs) genes, complete cds
HQ830479.1 Oropouche virus isolate H758669 nucleoprotein (N) and non-structural protein (NSs) genes, complete cds
HQ830478.1 Oropouche virus isolate H758687 nucleoprotein (N) and non-structural protein (NSs) genes, complete cds
HQ830477.1 Oropouche virus isolate H708717 nucleoprotein (N) and non-structural protein (NSs) genes, complete cds
HQ830476.1 Oropouche virus isolate H707157 nucleoprotein (N) and non-structural protein (NSs) genes, complete cds
HQ830475.1 Oropouche virus isolate H708139 nucleoprotein (N) and non-structural protein (NSs) genes, complete cds
HQ830474.1 Oropouche virus isolate H706893 nucleoprotein (N) and non-structural protein (NSs) genes, complete cds
HQ830473.1 Oropouche virus isolate H706890 nucleoprotein (N) and non-structural protein (NSs) genes, complete cds
HQ830472.1 Oropouche virus isolate H543880 nucleoprotein (N) and non-structural protein (NSs) genes, complete cds
HQ830471.1 Oropouche virus isolate H543857 nucleoprotein (N) and non-structural protein (NSs) genes, complete cds
HQ830470.1 Oropouche virus isolate H543760 nucleoprotein (N) and non-structural protein (NSs) genes, complete cds
HQ830469.1 Oropouche virus isolate H543639 nucleoprotein (N) and non-structural protein (NSs) genes, complete cds
HQ830468.1 Oropouche virus isolate H543638 nucleoprotein (N) and non-structural protein (NSs) genes, complete cds
Q830467.1 Oropouche virus isolate H543629 nucleoprotein (N) and non-structural protein (NSs) genes, complete cds
HQ830466.1 Oropouche virus isolate H543100 nucleoprotein (N) and non-structural protein (NSs) genes, complete cds
HQ830462.1 Oropouche virus isolate H532422 nucleoprotein (N) and non-structural protein (NSs) genes, complete cds
HQ830461.1 Oropouche virus isolate H532314 nucleoprotein (N) and non-structural protein (NSs) genes, complete cds
HQ830460.1 Oropouche virus isolate H505805 nucleoprotein (N) and non-structural protein (NSs) genes, complete cds
HQ830459.1 Oropouche virus isolate H505768 nucleoprotein (N) and non-structural protein (NSs) genes, complete cds
HQ830458.1 Oropouche virus isolate H505764 nucleoprotein (N) and non-structural protein (NSs) genes, complete cds
HQ830457.1 Oropouche virus isolate H498913 nucleoprotein (N) and non-structural protein (NSs) genes, complete cds
HQ830455.1 Oropouche virus isolate H472433 nucleoprotein (N) and non-structural protein (NSs) genes, complete cds
HQ830448.1 Oropouche virus isolate AR366927 nucleoprotein (N) and non-structural protein (NSs) genes, complete cds
HQ830445.1 Oropouche virus isolate H271078 nucleoprotein (N) and non-structural protein (NSs) genes, complete cds

HQ830443.1 Oropouche virus isolate AR136921 nucleoprotein (N) and non-structural protein (NSs) genes, complete cds
EF467372.1 Oropouche virus strain PMOH 682431 nucleocapsid protein (N) and non-structural protein (NSs) genes, complete cds
EF467371.1 Oropouche virus strain PMOH 682426 nucleocapsid protein (N) and non-structural protein (NSs) genes, complete cds
EF467370.1 Oropouche virus strain PPS 522 H 669314 nucleocapsid protein (N) and non-structural protein (NSs) genes, complete cds
EF467369.1 Oropouche virus strain PPS 523 H 669315 nucleocapsid protein (N) and non-structural protein (NSs) genes, complete cds
EF467368.1 Oropouche virus strain BeH 622544 nucleocapsid protein (N) and non-structural protein (NSs) genes, complete cds
AY704568.1 Oropouche virus strain BeH 543760 structural nucleocapsid protein (N) and non-structural protein (NSs) mRNAs, complete cds
AY704567.1 Oropouche virus strain BeH 543790 structural nucleocapsid protein (N) and non-structural protein (NSs) mRNAs, complete cds
AY704566.1 Oropouche virus strain BeH 543857 structural nucleocapsid protein (N) and non-structural protein (NSs) mRNAs, complete cds
AY704565.1 Oropouche virus strain BeH 543880 structural nucleocapsid protein (N) and non-structural protein (NSs) mRNAs, complete cds
AY704564.1 Oropouche virus strain BeH 543638 structural nucleocapsid protein (N) and non-structural protein (NSs) mRNAs, complete cds
AY704563.1 Oropouche virus strain BeH 543629 structural nucleocapsid protein (N) and non-structural protein (NSs) mRNAs, complete cds
AY704562.1 Oropouche virus strain BeH 543639 structural nucleocapsid protein (N) and non-structural protein (NSs) mRNAs, complete cds
AY704561.1 Oropouche virus strain BeH 543745 structural nucleocapsid protein (N) and non-structural protein (NSs) mRNAs, complete cds
AY704560.1 Oropouche virus strain BeH 543733 structural nucleocapsid protein (N) and non-structural protein (NSs) mRNAs, complete cds
AY704559.1 Oropouche virus strain BeH 521086 structural nucleocapsid protein (N) and non-structural protein (NSs) mRNAs, complete cds
AY237111.1 Oropouche virus segment S, complete sequence
AY117135.3 Oropouche virus strain BeAn 626990 segment S, complete sequence
AY993912.1 Oropouche virus isolate BeAn 208823 nucleocapsid protein and non-structural protein mRNAs, complete cds
AY993911.1 Oropouche virus isolate BeAn 208819 nucleocapsid protein and non-structural protein mRNAs, complete cds
AY993910.1 Oropouche virus isolate BeAn 208402 nucleocapsid protein and non-structural protein mRNAs, complete cds
AY993909.1 Oropouche virus isolate BeAn 206119 nucleocapsid protein and non-structural protein mRNAs, complete cds
AF164558.1 Oropouche virus strain GML450093 small segment nucleoprotein gene, partial cds; and non-structural protein (NSs) gene, complete cds
AF164557.1 Oropouche virus strain GML445252 small segment nucleoprotein gene, partial cds; and non-structural protein (NSs) gene, complete cds
AF164556.1 Oropouche virus strain GML444911 small segment nucleoprotein gene, partial cds; and non-structural protein (NSs) gene, complete cds
AF164555.1 Oropouche virus strain GML444477 small segment nucleoprotein gene, partial cds; and non-structural protein (NSs) gene, complete cds
AF164554.1 Oropouche virus strain IQT7085 small segment nucleoprotein gene, partial cds; and non-structural protein (NSs) gene, complete cds
AF164553.1 Oropouche virus strain 01-812-98 small segment nucleoprotein gene, partial cds; and non-structural protein (NSs) gene, complete cds
AF164552.1 Oropouche virus strain IQT4083 small segment nucleoprotein gene, partial cds; and non-structural protein (NSs) gene, complete cds
AF164551.1 Oropouche virus strain DEI209 small segment nucleoprotein gene, partial cds; and non-structural protein (NSs) gene, complete cds
AF164550.1 Oropouche virus strain MD023 small segment nucleoprotein gene, partial cds; and non-structural protein (NSs) gene, complete cds
AF164549.1 Oropouche virus strain IQT1690 small segment nucleoprotein gene, partial cds; and non-structural protein (NSs) gene, complete cds
AF164548.1 Oropouche virus strain BeH543618 small segment nucleoprotein gene, partial cds; and non-structural protein (NSs) gene, complete cds
AF164547.1 Oropouche virus strain BeH543087 small segment nucleoprotein gene, partial cds; and non-structural protein (NSs) gene, complete cds
AF164546.1 Oropouche virus strain BeH544552 small segment nucleoprotein gene, partial cds; and non-structural protein (NSs) gene, complete cds
AF164545.1 Oropouche virus strain BeH543033 small segment nucleoprotein gene, partial cds; and non-structural protein (NSs) gene, complete cds
AF164544.1 Oropouche virus strain BeH541863 small segment nucleoprotein gene, partial cds; and non-structural protein (NSs) gene, complete cds
AF164543.1 Oropouche virus strain BeH505663 small segment nucleoprotein gene, partial cds; and non-structural protein (NSs) gene, complete cds
AF164542.1 Oropouche virus strain BeH505442 small segment nucleoprotein gene, partial cds; and non-structural protein (NSs) gene, complete cds
AF164541.1 Oropouche virus strain BeH504514 small segment nucleoprotein gene, partial cds; and non-structural protein (NSs) gene, complete cds
AF164540.1 Oropouche virus strain BeH475248 small segment nucleoprotein gene, partial cds; and non-structural protein (NSs) gene, complete cds
AF164539.1 Oropouche virus strain BeAr473358 small segment nucleoprotein gene, partial cds; and non-structural protein (NSs) gene, complete cds
AF164538.1 Oropouche virus strain BeH472204 small segment nucleoprotein gene, partial cds; and non-structural protein (NSs) gene, complete cds
AF164537.1 Oropouche virus strain BeH472200 small segment nucleoprotein gene, partial cds; and non-structural protein (NSs) gene, complete cds
AF164536.1 Oropouche virus strain BeH390233 small segment nucleoprotein gene, partial cds; and non-structural protein (NSs) gene, complete cds
AF164535.1 Oropouche virus strain BeH381114 small segment nucleoprotein gene, partial cds; and non-structural protein (NSs) gene, complete cds
AF164534.1 Oropouche virus strain BeH379693 small segment nucleoprotein gene, partial cds; and non-structural protein (NSs) gene, complete cds
AF164533.1 Oropouche virus strain BeH271815 small segment nucleoprotein gene, partial cds; and non-structural protein (NSs) gene, complete cds
AF164532.1 Oropouche virus strain BeAn19991 small segment nucleoprotein gene, partial cds; and non-structural protein (NSs) gene, complete cds

Other Bunyviruses with 100% match

KP691629.1 Perdoes virus isolate BeAn790177 segment S, complete sequence
KJ866386.1 Iquitos virus strain MIS-0397 nucleocapsid protein and nonstructural protein genes, complete cds
KF697146.1 Madre de Dios virus isolate FMD 1303 nucleocapsid protein gene, complete cds
KF697144.1 Iquitos virus isolate IQT9924 nucleocapsid protein gene, complete cds
JQ675601.5 Jatobal virus nucleocapsid protein and nonstructural protein genes, complete cds
KF150535.1 Iquitos virus strain IQE3728/PER/06 nucleoprotein gene, partial cds
KF150525.1 Iquitos virus strain IQE1704/PER/05 nucleoprotein gene, partial cds
KF150523.1 Iquitos virus strain IQE1217/PER/05 nucleoprotein gene, partial cds

KF150522.1 Iquitos virus strain IQE1005/PER/05 nucleoprotein gene, partial cds
AF312382.1 Jatobal virus nucleoprotein (N) gene, partial cds; and non-structural protein (NSs) gene, complete cds

1 mismatch forward primer

KP026181.1 Oropouche virus strain TRVL9760 segment S, complete sequence
KJ866389.1 Madre de Dios virus strain INHRR 17a-10 structural nucleocapsid protein and nonstructural protein genes, complete cds
KC759124.1 Oropouche virus strain TRVL-9760 segment S nucleocapsid protein (N) and nonstructural protein (NS) genes, complete cds
HM470120.1 Oropouche virus strain BeH384193 nucleocapsid protein gene, partial cds
HM470119.1 Oropouche virus strain BeH384192 nucleocapsid protein gene, partial cds
HM470106.1 Oropouche virus strain BeH472435 nucleocapsid protein gene, partial cds
HM470107.1 Oropouche virus strain BeAr19886 nucleocapsid protein gene, complete cds
HQ830487.1 Oropouche virus isolate H759023 nucleoprotein (N) and non-structural protein (NSs) genes, complete cds
HQ830456.1 Oropouche virus isolate H472435 nucleoprotein (N) and non-structural protein (NSs) genes, complete cds
HQ830451.1 Oropouche virus isolate H384193 nucleoprotein (N) and non-structural protein (NSs) genes, complete cds
HQ830450.1 Oropouche virus isolate H384192 nucleoprotein (N) and non-structural protein (NSs) genes, complete cds
AF164531.1 Oropouche virus strain TRVL9760 small segment nucleoprotein gene, partial cds; and non-structural protein (NSs) gene, complete cds

1 mismatch reverse primer

HM470112.1 Oropouche virus strain BeH244576 nucleocapsid protein gene, partial cds
HM470104.1 Oropouche virus strain BeH390242 nucleocapsid protein gene, partial cds
HM470103.1 Oropouche virus strain BeH389865 nucleocapsid protein gene, partial cds
HM470126.1 Oropouche virus strain BeH541140 nucleocapsid protein gene, partial cds
HQ830482.1 Oropouche virus isolate H759531 nucleoprotein (N) and non-structural protein (NSs) genes, complete cds
HQ830454.1 Oropouche virus isolate H390242 nucleoprotein (N) and non-structural protein (NSs) genes, complete cds
HQ830453.1 Oropouche virus isolate H389865 nucleoprotein (N) and non-structural protein (NSs) genes, complete cds
HQ830444.1 Oropouche virus isolate H244576 nucleoprotein (N) and non-structural protein (NSs) genes, complete cds
HM470110.1 Oropouche virus strain BeH121923 nucleocapsid protein gene, complete cds
HM470109.1 Oropouche virus strain BeH29090 nucleocapsid protein gene, complete cds
HM470108.1 Oropouche virus strain BeH29086 nucleocapsid protein gene, complete cds

1 mismatch in both forward and reverse primers

HM470124.1 Oropouche virus strain BeH532490 nucleocapsid protein gene, partial cds
HM470121.1 Oropouche virus strain BeH385591 nucleocapsid protein gene, partial cds
HM470118.1 Oropouche virus strain BeH366781 nucleocapsid protein gene, partial cds
HM470116.1 Oropouche virus strain BeH356898 nucleocapsid protein gene, partial cds
HQ830480.1 Oropouche virus isolate H759525 nucleoprotein (N) and non-structural protein (NSs) genes, complete cds
HQ830463.1 Oropouche virus isolate H532490 nucleoprotein (N) and non-structural protein (NSs) genes, complete cds
HQ830452.1 Oropouche virus isolate H385591 nucleoprotein (N) and non-structural protein (NSs) genes, complete cds
HQ830449.1 Oropouche virus isolate H366781 nucleoprotein (N) and non-structural protein (NSs) genes, complete cds
HQ830447.1 Oropouche virus isolate H356898 nucleoprotein (N) and non-structural protein (NSs) genes, complete cds

2 mismatch forward primer, 1 reverse primer

HM470125.1 Oropouche virus strain BeH532500 nucleocapsid protein gene, partial cds
HM470101.1 Oropouche virus strain BeH543091 nucleocapsid protein gene, partial cds
HQ830465.1 Oropouche virus isolate H543091 nucleoprotein (N) and non-structural protein (NSs) genes, complete cds
HQ830464.1 Oropouche virus isolate H532500 nucleoprotein (N) and non-structural protein (NSs) genes, complete cds

MAYV 100% match

KT754168.1 Mayaro virus isolate BeAr20290, complete genome
KP842820.1 Mayaro virus strain BeAr30853 Nsp1-3, Nsp4, and structural polyprotein genes, complete cds
KP842819.1 Mayaro virus strain BeH256 Nsp1-3, Nsp4, and structural polyprotein genes, complete cds
KP842818.1 Mayaro virus strain BeAr505411 Nsp1-3, Nsp4, and structural polyprotein genes, complete cds
KT818520.1 Mayaro virus isolate BR/SJRP/LPV01/2015, complete genome
KJ879258.1 Mayaro virus isolate MAYV_BR/MT_VG22/2012 nonstructural protein 1 gene, partial cds
KJ879257.1 Mayaro virus isolate MAYV_BR/MT_CBA308/2012 nonstructural protein 1 gene, partial cds
KJ879256.1 Mayaro virus isolate MAYV_BR/MT_CBA306/2012 nonstructural protein 1 gene, partial cds

KJ879255.1 Mayaro virus isolate MAYV_BR/MT_NSL12/2012 nonstructural protein 1 gene, partial cds
KJ713282.1 Mayaro virus strain MAYV_BR/MT_VG20/2012 non-structural protein 1 gene, partial cds
KF305672.1 Mayaro virus isolate MT/SINOP/210/2011 nonstructural protein 1 gene, partial cds
AY348561.1 Mayaro virus nonfunctional nonstructural protein 1 (nsp1) gene, partial sequence
DQ138319.1 Mayaro virus isolate BeAr505411 nonstructural protein 1 gene, partial cds
AF237947.1 Mayaro virus, complete genome
KJ739869.1 Mayaro virus strain MAYV_BR/MT_VG147/2012 non-structural protein 1 gene, partial cds
KP842816.1 Mayaro virus strain FPI0179 Nsp1-3, Nsp4, and structural polyprotein genes, complete cds
KP842815.1 Mayaro virus strain FPI1761 Nsp1-3, Nsp4, and structural polyprotein genes, complete cds
KP842813.1 Mayaro virus strain FPY0046 Nsp1-3, Nsp4, and structural polyprotein genes, complete cds
KP842810.1 Mayaro virus strain TRVL15537 Nsp1-3, Nsp4, and structural polyprotein genes, complete cds
KP842809.1 Mayaro virus strain BeH186258 Nsp1-3, Nsp4, and structural polyprotein genes, complete cds
KP842808.1 Mayaro virus strain IQU3056 Nsp1-3, Nsp4, and structural polyprotein genes, complete cds
KP842807.1 Mayaro virus strain Ohio Nsp1-3, Nsp4, and structural polyprotein genes, complete cds
KP842801.1 Mayaro virus strain IQE2777 Nsp1-3, Nsp4, and structural polyprotein genes, complete cds
KP842800.1 Mayaro virus strain ARV0565 Nsp1-3, Nsp4, and structural polyprotein genes, complete cds
KP842799.1 Mayaro virus strain MAYV15A Nsp1-3, Nsp4, and structural polyprotein genes, complete cds
KP842798.1 Mayaro virus strain MAYV14A Nsp1-3, Nsp4, and structural polyprotein genes, complete cds
KP842797.1 Mayaro virus strain MAYV13A Nsp1-3, Nsp4, and structural polyprotein genes, complete cds
KP842796.1 Mayaro virus strain MAYV12A Nsp1-3, Nsp4, and structural polyprotein genes, complete cds
KP842795.1 Mayaro virus strain MAYV11A Nsp1-3, Nsp4, and structural polyprotein genes, complete cds
KP842794.1 Mayaro virus strain MAYV16A Nsp1-3, Nsp4, and structural polyprotein genes, complete cds
KJ013266.2 Mayaro virus strain BNI-1, complete genome
KJ879259.1 Mayaro virus isolate MAYV_BR/MT_CBA301/2012 nonstructural protein 1 gene, partial cds
KJ870987.1 Mayaro virus strain MAYV_BR/MT_CBA322/2012 nonstructural protein 1 gene, partial cds
DQ138318.1 Mayaro virus isolate Uruma nonstructural protein 1 gene, partial cd
DQ138316.1 Mayaro virus isolate OBS2248 nonstructural protein 1 gene, partial cds
DQ138315.1 Mayaro virus isolate BeH504378 nonstructural protein 1 gene, partial cds
U94602.1 Mayaro virus nonstructural protein 1 (nsP1) gene, partial cds

1 mismatch

KJ879254.1 Mayaro virus isolate MAYV_BR/MT_CBA305/2012 nonstructural protein 1 gene, partial cds
KJ682316.1 Mayaro virus strain MAYV_BR/MT_CBA9/2012 non-structural protein 1 gene, partial cds
KP842817.1 Mayaro virus strain FVB0069 Nsp1-3, Nsp4, and structural polyprotein genes, complete cds
KP842814.1 Mayaro virus strain FVB0112 Nsp1-3, Nsp4, and structural polyprotein genes, complete cds
KP842812.1 Mayaro virus strain FMD3213 Nsp1-3, Nsp4, and structural polyprotein genes, complete cds
KP842811.1 Mayaro virus strain FMD0641 Nsp1-3, Nsp4, and structural polyprotein genes, complete cds
KP842806.1 Mayaro virus strain FSB1131 Nsp1-3, Nsp4, and structural polyprotein genes, complete cds
KP842805.1 Mayaro virus strain FSB0319 Nsp1-3, Nsp4, and structural polyprotein genes, complete cds
KM400591.1 Mayaro virus strain Acre27, complete genome
KJ879253.1 Mayaro virus isolate MAYV_BR/MT_CBA230/2012 nonstructural protein 1 gene, partial cds
KJ879333.1 Mayaro virus isolate MAYV_BR/MT_SOR246/2012 nonstructural protein 1 gene, partial cds
DQ001069.1 Mayaro virus strain MAYLC from French Guiana, complete genome
